# An Elegant Solution to a Ruptured Right Aberrant Subclavian Artery after Oesophageal Stent Removal

**DOI:** 10.1155/2021/8891012

**Published:** 2021-04-10

**Authors:** Daniel Thompson, Sophie Cerutti, Muhammad Peerbux, Anna Ikponmwosa, Hansraj Bookun, Yahya Lahham

**Affiliations:** Department of Vascular Surgery. St. Vincent's Hospital Melbourne, Australia

## Abstract

Arterioenteric or arteriotracheal fistula is a known complication of an aberrant right subclavian artery (ARSA) and is often associated with prolonged nasogastric or endotracheal intubation or oesophageal stenting. Fistula formation from the ARSA can present unexpectedly with rapid exsanguination with massive haemoptysis or haematemesis, and unless promptly recognised and treated is rapidly fatal. We present a novel endovascular method for treating a fistula between the oesophagus, trachea and an ARSA in an unstable patient following oesophageal stent removal, utilising a covered iliac limb stent, eliminating the need for an open surgical approach.

## 1. Case Report

An aberrant right subclavian artery (ARSA) is a common aortic arch anomaly, with an incidence of 0.5-1% [1, 2]. Embryologically, it is caused by regression of the fourth aortic arch between the carotid and subclavian arteries [1–3] to originate distal to the left subclavian artery origin, coursing behind the oesophagus [2, 4]. Arterioenteric fistula is a rare complication with mortality upwards of 70% [2].

A 72-year-old man with hypertension presented with haematemesis and a syncopal episode, 1 month after Ivor Lewis oesophagectomy for the management of an oesophageal squamous cell carcinoma. He had a 23 mm × 105 mm Boston Scientific oesophageal stent to address an anastomotic stricture and a tracheoesophageal fistula.

**Figure 1:**
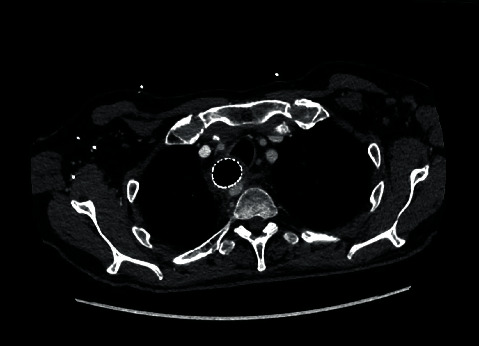
Axial CT angiogram slice demonstrating ARSA crossing behind the oesophagus with oesophageal stent in situ.

CT-angiography demonstrated no active bleeding and an ARSA abutting the oesophagus (Figure 1).

A haemoglobin drop from 104 g/L to 57 g/L requiring transfusion of four units of packed red blood cells. He underwent endoscopic evaluation where fresh blood in the oesophageal lumen was found. The oesophageal stent was removed, and this resulted in torrential bleeding from the patient's mouth and endotracheal tube. He had a cardiac arrest and required cardiopulmonary resuscitation.

A Sengstaken-Blakemore tube was placed without improvement. Spontaneous return of circulation was achieved after a CRE PRO oesophageal balloon (Boston Scientific, USA) was inflated. The vascular surgery team was contacted, and he was moved to the hybrid-operating suite.

**Figure 2:**
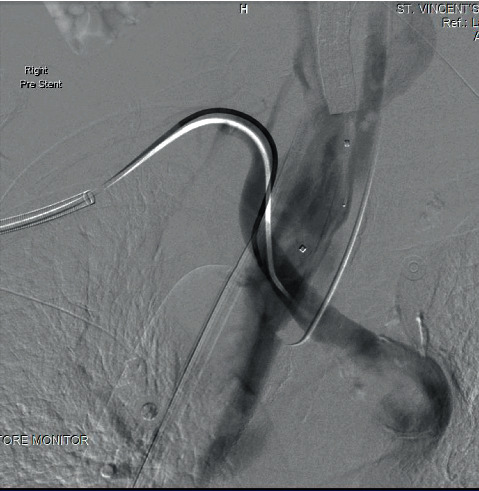
DSA angiography with oesophageal balloon deflated showing massive oesophageal haemorrhage from the ARSA.

**Figure 3:**
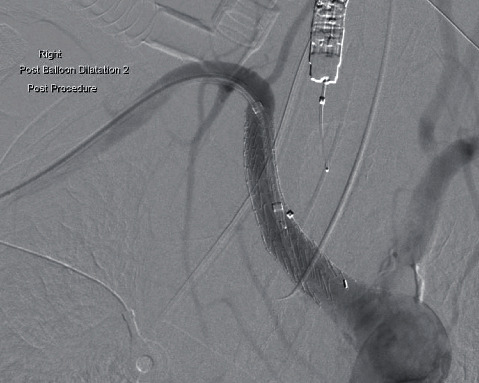
Completion DSA angiography demonstrating forward flow of contrast without extravasation through the Gore 16 × 10 × 70 mm iliac stent deployed in the ARSA.

A right brachial artery cutdown to insert a long 12Fr sheath was performed. Diagnostic angiography with the oesophageal balloon deflated demonstrated active bleeding from the ARSA (Figure 2). A tapered GORE-covered iliac stent (16 mm × 10 mm × 70 mm) was deployed from the proximal ARSA across the bleeding point with the oesophageal balloon inflated. The stent was postdilated with a 32 mm CODA moulding balloon (Cook Medical). Completion angiography with the oesophageal balloon deflated demonstrated forward flow in the subclavian artery, a patent vertebral artery, and no extravasation of contrast (Figure 3). He was extubated on the next day and commenced on lifelong antibiotics.

## 2. Discussion

Arterioenteric fistula is a rare complication of ARSA, most commonly associated with prolonged nasogastric (NGT) or endotracheal intubation (ETT), or presence of an oesophageal stent [2]. Diagnosis is difficult as patients present with rapid unexplained exsanguination with overwhelming haematemesis. Descriptions of nonaneurysmal ARSA-oesophageal fistula are limited to case reports, and there is no consensus on the surgical approach.

The initial management priority is aggressive patient resuscitation, followed by attempted tamponade of the bleeding. Multiple authors describe temporary endoscopic control of bleeding with a Sengstaken-Blakemore tube or esophageal balloon. Endovascular approaches offer the possibility for intraluminal balloon tamponade [5]. When these techniques are not possible, the final option is for thoracotomy or sternotomy to establish proximal arterial control via cross clamping of the aorta [6]. We describe the first case in the literature of an ARSA-esophageal fistula managed endovascularly without thoracotomy or sternotomy [2].

Hybrid surgical approaches utilize open arterial access, selective angiography, and stenting with either self-expanding or balloon-expandable covered stents such as VIABAHN (Gore, Arizona USA) or Atrium V12 (Getinge, Sweden) [7, 8]. Embolization of the ARSA stump is common, following open ligation and revascularization. It is preferable to maintain patency of the vertebral artery, as coverage may precipitate posterior circulation stroke.

Open surgical techniques include thoracotomy or median sternotomy with ligation of the proximal ARSA and carotid to subclavian bypass grafting or aortoaxillary bypass grafting [2, 7, 9, 10].

The predominant learning point of this case is to caution in the placement and removal of oesophageal stents in patients with known ARSA. Previous authors have recommended avoiding long-term nasogastric or endotracheal intubation in patients with known ARSA, as they are a risk factor for fistula development and rupture [5]. We recommend the same caution prior to placement of oesophageal stents in patients with known ARSA. Most patients with oesophageal stents will have CT imaging which should demonstrate the presence of ARSA prior to insertion and/or removal. In patients in whom oesophageal stenting cannot be avoided, removal should occur in a hybrid operating suite with the availability of cardiothoracic and vascular surgical teams. Coordination of manoeuvres between the endoscopist and the vascular surgical team was critical in this case.

This case highlights the unique challenges of dealing with exsanguinating haemorrhage in the setting of aberrant anatomy and recent complex operative interventions for oesophageal cancer.
